# Enhancing Gene Synthesis Security: An Updated Framework for Synthetic Nucleic Acid Screening and the Responsible Use of Synthetic Biological Materials

**DOI:** 10.1089/apb.2023.0036

**Published:** 2024-06-20

**Authors:** Curtis Matthew Sharkey, Mariam Lekveishvili, Tricia de la Rosa, Katheen Danskin

**Affiliations:** ^1^ Strategy Division, Office of Strategy, Policy, and Requirements, Administration for Strategic Preparedness and Response, U.S. Department of Health and Human Services, Washington, District of Columbia, USA.; ^2^ Thriving Environments, San Diego, California, USA.

**Keywords:** Screening Framework Guidance, biosecurity, synthetic dsDNA, synthetic nucleic acids, sequences of concern

## Abstract

**Introduction:**

In response to continuous advancements in synthetic biotechnologies and in the availability of synthetic nucleic acids to the biological research community since the publication of the 2010 HHS synthetic double-stranded DNA (dsDNA) screening framework, the U.S. government undertook a comprehensive and stakeholder-driven review and revision process. This culminated in the publication of a new screening framework for synthetic nucleic acids in October 2023, followed by an Executive Order directing departments and agencies of the U.S. government to take certain measures in support of implementing the screening framework. This review provides an overview of the process by which stakeholder comments were considered and by which the 2023 screening framework was drafted. A summary of expected impacts on the life sciences research community is also provided.

**Methods:**

Comments were solicited from synthetic biology stakeholders through the publication of two *Federal Register* Notices, in 2020 and 2022. The 2020 Notice elicited 15 unique responses totaling 220 pages, and the 2022 Notice elicited 26 unique responses totaling 79 pages. These were considered by a deliberative interagency group, resulting in a revised screening framework in 2023.

**Discussion and Conclusion:**

The adoption of the 2023 screening framework, and related provisions in the Executive Order that followed, will impact researchers and biosafety officers across the U.S. bioeconomy. For instance, this screening framework is no longer limited in its recommendations to providers of synthetic dsDNA containing sequences unique to regulated pathogens or toxins, but now includes recommendations to all entities involved in the sale, use, and transfer of all forms of synthetic nucleic acids encoding genetic sequences that contribute to pathogenicity or toxicity—whether from regulated agents or not. Biosafety professionals are emerging as a critical resource for establishing and fostering a culture of biosecurity surrounding synthetic nucleic acids containing these high consequence genetic sequences.

**Significance:**

The work presented is significant because the scope of the 2010 screening framework has been expanded to include roles and responsibilities for new entities across the life sciences research landscape. This will likely impact biosafety professionals, who may be well positioned in their institutions to coordinate these new responsibilities.

## Introduction

Synthetic biology is an interdisciplinary field that focuses on both the design and fabrication of novel biological components and systems as well as the redesign and fabrication of existing biological systems. It has transformed technologies in the life sciences and beyond. As synthetic biology can be used to generate pathogenic sequences and strains de novo, it has the potential to be used to create existing and novel organisms, including pathogens, which could threaten public health, agriculture, plants, animals, animal and plant products, and the environment.

For instance, synthetic nucleic acids can be ordered online, and this material can be used to create viral genomes, using molecular techniques that have become more widely available. This increasingly obviates the need for access to the naturally occurring agents or naturally occurring genetic material from these agents. The potential availability of dangerous agents has thereby been greatly expanded.

In the United States the possession, use, and transfer of certain pathogens and toxins are restricted by the Federal Select Agents Program (FSAP),^1^ and the international shipment of specified biological agents are restricted by the Export Administration Regulations' Commerce Control List (CCL).^2^ To reduce the risk that unauthorized individuals might exploit the application of double-stranded DNA (dsDNA) synthesis technology to obtain restricted genetic materials, the U.S. government issued the *Screening Framework Guidance for Providers of Synthetic Double-Stranded DNA* in 2010.^3^

This set forth recommended baseline standards for the gene and genome synthesis industry and other providers of synthetic dsDNA products regarding screening orders and verifying customer legitimacy when dsDNA orders contain sequences unique to regulated agents (i.e., by FSAP or CCL).

Since the 2010 guidance was issued, there have been remarkable advances in synthetic biology. Polio virus, which has a small positive-sense single-stranded RNA (+ssRNA) genome, of about 7500 nucleotides (nt), was rescued from chemically synthesized cDNA in 2001.^4^ At that time, it was presumed that most especially dangerous pathogens, including orthopoxviruses, had genomes too large and complicated to be synthesized de novo.

However, in 2017, Canadian researchers reported success in generating a replicative orthopoxvirus (horsepox virus) using only synthetic dsDNA that they had ordered from a company in the United States.^5,6^ One of the most access-controlled pathogens on the planet is also an orthopoxvirus—variola virus, the etiological agent of smallpox. By international agreement, variola virus is only possessed by one laboratory in Russia and one in the United States.

When it became clear that the synthesis of orthopoxviruses was possible using synthetic nucleic acids, the U.S. government reconsidered the adequacy of the 2010 screening framework. The 2010 recommendations were for companies selling genetic materials to screen each 200 base pair (bp) window in submitted orders for sequences that are a best match (using the Basic Local Alignment Search Tool [BLAST],^7^ or a similar gene sequence alignment platform) to a regulated agent but not to a nonregulated one.

Comparing horsepox virus' genomic sequence to either variola major or minor viruses reveals several 200 bp regions that are completely shared between these pathogens (e.g., 214 bp between 96942–97156 of horsepox virus and 76415–76629 of variola virus).^8,9^ However, as the shared sequences are not unique to variola virus, even a company diligently following the 2010 recommendations would have no obligation to investigate further when these regions of shared identity are present in an order.

In addition to emerging capabilities for synthesizing or engineering increasingly complicated pathogens, it is also possible to introduce synthetic genes into nonpathogenic organisms or unregulated pathogens to make them similarly pathogenic or toxic as those that are regulated by FSAP or CCL. For instance, there are publicly available databases of genetic sequences that have been experimentally determined to contribute to pathogenicity or toxicity.^10^

These databases are hard to track and pose an increasing public health threat, providing a potential access point for individuals with malicious intent—or without the necessary competence to safely handle recombinant pathogens—to create a recombinant especially dangerous pathogen. In 2017, the HHS Administration for Strategic Preparedness and Response (ASPR) convened an interagency working group to assess these emerging threats and mitigate the risks that they pose through revisions to the 2010 screening framework.

## Methods

To ensure that any updates to the 2010 screening framework incorporated the viewpoints of experts in the academic and industrial sectors, ASPR collected stakeholder feedback through a 2020 *Federal Register* Notice, titled *Review and Revision of the Screening Framework Guidance for Providers of Synthetic Double-Stranded DNA*,^11^ and, based on 15 unique comments totaling 220 pages, undertook a deliberative process with its interagency colleagues to propose updates to the existing guidance. In 2022, a revised draft screening framework was published as another *Federal Register* Notice, titled *Screening Framework Guidance for Providers and Users of Synthetic Oligonucleotides*,^12^ to solicit stakeholder feedback.

After a second round of deliberations on stakeholder feedback, which elicited 26 unique comments totaling 79 pages, the screening framework was updated in ways that address the changing biotechnology landscape (Table 1). The new screening framework is intended to be forward-looking; although most recommendations are implementable with current technology, some are “best practices” that should be worked toward as soon as it is practical to do so. Using this framing will give the screening framework longevity and allows for some flexibility as screening technologies advance.

**Table 1.** Significant updates to the 2023 screening framework that will potentially impact business operations by all entities involved in the provision, use, and transfer of synthetic nucleic acids containing sequences of concern and benchtop nucleic acid synthesizers

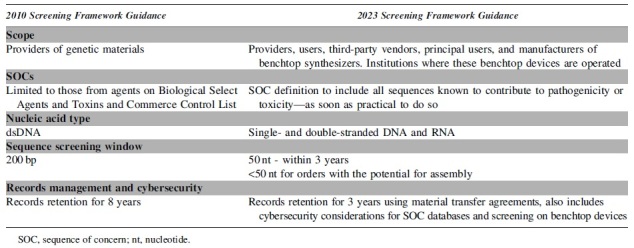

This review summarizes how public comments from 2020 to 2022 elicited certain considerations in the 2023 *Screening Framework Guidance for Providers and Users of Synthetic Nucleic Acids*,^13^ and outlines the justification for updates to it. The full comments received in response to the 2020 and 2022 *Federal Register* Notices are available on the ASPR website,^14,15^ and the summary of changes in this review has been adapted from a summary of changes between the 2010 screening framework and the 2022 *Federal Register* Notice of a proposed updated screening framework—which was posted to the ASPR website in 2022.^16^

## Scope of the Guidance

Most stakeholder responses agreed on extending the scope of the new guidance beyond the FSAP and CCL agents. However, some commenters also mentioned that if the scope were expanded, there might be negative impacts, including increased cost/burden of screening and potential negative effects on research, the bioeconomy, and implementation by small businesses. Responses centered on methods to define a sequence of concern (SOC) in a manner that enables consideration of sequences that may pose biosecurity risks, but that are not included in the FSAP and CCL lists.

Although some responses posed well developed and comprehensive mechanisms to curate inclusion of annotated sequences into a database of SOCs and assess whether the compilation of those sequences should pose restrictions on an order, there was not unanimity in whether this approach would be practical given the curation needs of databases, inadequate current infrastructure, and the immaturity of approaches to predict functionality of genetic sequences.

Stakeholders also noted that expanding beyond a defined list of SOCs may make the screening easier, since it can be difficult to apply certain criteria in the 2010 screening framework that exclude housekeeping genes from being SOCs, even when they are from agents on the FSAP or CCL lists. As making the determination about these types of housekeeping genes is not always trivial, stakeholders reported that it is the main source of the added cost of follow-up screening.

This can require detailed knowledge of the function of sequences that would be included in the schema suggested for expanding beyond list-based approaches. Most respondents also thought that the screening framework should be expanded to other providers in the supply chain, such as third-party sellers or others involved in the transfer SOCs beyond the initial purchaser.

As a result of these suggestions and concerns, the definition of SOCs in the 2023 screening framework has been expanded to include both sequences from agents in the FSAP or CCL lists and sequences that contribute to the pathogenicity or toxicity of agents not included in these lists. Furthermore, to fully encompass the risks to national security that may be associated with the misuse of synthetic biology, the SOC definition has been expanded to be inclusive of pathogenicity or toxicity that threatens public health agriculture, plants, animals, animal products, or the environment.

Although this is a dramatically expanded scope for sequences that should be handled with enhanced care by responsible entities, these changes were made to encompass the suggestions made by stakeholders and will aid synthetic nucleic acid providers and customers in helping to ensure biosecurity. The identification of SOCs in orders also has been democratized in the new screening framework, in that customers are encouraged to preemptively provide evidence of their legitimacy when placing orders that they are aware contain SOCs.

Customers almost always will be aware of whether the sequence of the nucleic acids that they are ordering is from a gene known to contribute to pathogenicity or toxicity. This shift to shared responsibility will both alleviate some of the burden on companies selling synthetic nucleic acids and help to establish a shared culture of responsibility for the responsible use and transfer of these materials.

As part of this expansion of responsibilities to include customers in this new guidance, there is also a recommendation that customers verify the legitimacy of anyone to whom they transfer these materials or benchtop nucleic acid synthesizers. These expansions are intended to ensure these potentially dangerous materials are used by competent and well-intentioned people throughout their lifecycle.

## Sequence Screening Methodology

The majority of respondents reported that the 200 bp screening window should be reduced to allow smaller embedded sequences to be found. There was no clear consensus, however, regarding the optimal window size. Although multiple responses indicate that the screening window should be reduced to 40 − 50 bp, some commenters found that a 200 bp window is sufficient, and that reducing the size of the window should be subjected to cost/benefit analysis. Most respondents indicated that BLAST is an appropriate screening tool.

Regarding the identification of SOCs, the lack of a definitive database of biothreat sequences was identified as a gap. Proteomic and bioinformatics approaches to curate a database of biothreat sequences and potentially establishing new methodologies other than BLAST were suggested. As mentioned in the Scope section of this article, it was noted several times in stakeholder comments that such a curated and annotated SOC database could be used in determining the risk associated with an order. Furthermore, stakeholders noted on this topic that such a determination could result in tiered approaches to mitigating the risks associated with orders.

Regarding Best Match flagging, some stakeholders preferred to continue using Best Match with small modifications, whereas others suggested moving to signature-based detection or other approaches. An outcome-based approach was also suggested, where the government sets performance standards and then evaluates the screening methodology against them. Several responses indicated that, although predictive bioinformatic approaches are in development, they are likely not sufficiently mature on their own to identify SOCs.

A molecular biology-based biorisk approach was described by several respondents, some suggesting that this approach would benefit from a centralized U.S. government sequence screening database—an annotated database of SOCs—which would facilitate biorisk assessment of experiments and genetic constructs across multiple Federal biosecurity policies and research oversight mechanisms. Respondents also indicated that the lack of a curated database of biothreat sequences outside the FSAP and CCL lists makes the development of these approaches unlikely.

Several commenters supported the consideration of order batch size and indicated that the screening framework should include a discussion on the use of synthetic nucleic acid fragments of a certain size for assembly into longer constructs and that providers should monitor Best Matches both (1) across sequences within an order and (2) across orders from the same customer.

In response to these suggestions, the 2023 screening framework continues to recommend that providers use Best Match for identifying SOCs, but also notes that other screening approach(es) that the provider assesses to be equivalent or superior to the Best Match approach would also be appropriate. This consideration may include using customized databases or approaches that evaluate the biological risk associated with nonselect agents and toxins sequences or, for international orders, sequences not associated with items on the CCL.

Also, the 2023 screening framework acknowledges that providers may wish to consider developing solutions for determining which sequences from pathogens, regulated or unregulated, should not cause concern (i.e., a list of genes that pose no pathogenic or toxicity risk). Notably, a recommendation also is included that providers should screen all sequences ordered by an individual customer, using a short sequence alignment software package. Providers are advised to consider these orders as containing SOCs if the resulting ungapped alignment of any of a customer's orders is a Best Match to any SOC, and if these sequences are constructed to allow their ligation to form the SOC (i.e., overlaps are present to support the construction of a larger nucleic acid, which itself is an SOC).

## Biosecurity Measures

Many comments indicated that the maintenance and implementation of broader list-based approach(es) are now feasible and several curated SOC databases exist that have annotated the functionalities of pathogenicity and toxicity. It is noteworthy, however, that these databases generally do not cover the entirety of the expanded types of pathogenicity or toxicity described in the SOC definition (i.e., pathogenicity or toxicity that threatens public health, agriculture, plants, animals, animal products, or the environment).

Other respondents noted that no single universally recognized reference database exists. Respondents indicated that a curated database of sequences directly subject to regulatory control would be extremely valuable to providers, and some respondents suggested that this database should be actively maintained in perpetuity by the U.S. government—up to and including same-day updates coinciding with additions or removals of organisms on FSAP or CCL lists and/or periodic updates when genes contributing to pathogenicity or toxicity are identified and published.

Supplementing the Best Match approach with curated SOC databases and predictive tools was suggested by some respondents, but the negative aspects of curated databases and predictive tools that may underestimate the hazard of sequences not included in them was also noted. Respondents also noted limitations in implementing broader list-based approaches. For example, the lack of operationally trained biosecurity experts was noted as a gap in broadly implementing these methodologies, and workforce training was mentioned as a potential area for U.S. government investment.

In response to these suggestions and concerns, the 2023 screening framework includes sequence screening recommendations that do not rely upon the use of curated databases of sequences that meet the definition of SOCs. However, the use of such databases is also not precluded, should they become available. Furthermore, as part of implementation activities directed by the October 2023 Executive Order discussed hereunder, the U.S. government will develop standards against which such databases could be assessed for sufficiency.

The U.S. government encourages the development of these databases of sequences of concern as screening tools that could be improved as additional data become available. Furthermore, to relieve some of the burden of sequence screening, the screening framework has been updated to include the recommendation that customers notify providers if their nucleic acid orders contain SOCs, as discussed in the Customer Screening section that follows.

## Customer Screening

Several respondents recommended the consideration of methods to streamline customer screening. Prescreening, whitelisting, and the maintenance of a restricted list were suggested as potential responsibilities of the U.S. government, and the risks and burdens associated with the whitelisting, as well as blacklisting, were discussed in detail by the interagency group. Some respondents supported prescreening of the customer first, and others supported prescreening of the sequences first.

Some indicated that enhanced customer screening would require a registration program, and that whitelisting of customers may be helpful. Difficulty with tracking international orders was also noted. Although most comments did not indicate that the screening framework poses an undue burden, the marginal cost of screening was noted to have increased as the overall cost of nucleic acid synthesis has decreased. Many comments mentioned that manual review of false positive findings is the greatest cost of screening, due to the cost of expert staff.

To relieve some of the burden of follow-up screening when SOCs are identified in an order, the screening framework has been updated to include the recommendation that customers notify providers that their synthetic nucleic acid orders contain SOCs and also provide proof of their legitimacy when placing such orders. The screening framework also recommends that the same verification of legitimacy should take place when these materials are transferred to other researchers, and records of the transfer should be kept for at least 3 years.

In addition, the screening framework and its accompanying Companion Guide outline the types of information that will be helpful in verifying the legitimacy of recipients.^17^ Examples of information that will be helpful in verifying legitimacy are provided in Table 2. It is noteworthy that although some types of information, such as documentation of internal review and approval of the project/research, or evidence provided by the recipient's Responsible Official that the recipient is registered with FSAP, may provide evidence of legitimacy by themselves, other types of information will be useful in combination to verify legitimacy—such as institutional or corporate affiliation and name of an institutional biosafety officer, or publication history and research plan.

**Table 2.** Types of information recommended for verifying the legitimacy of sequences of concern customers or recipients

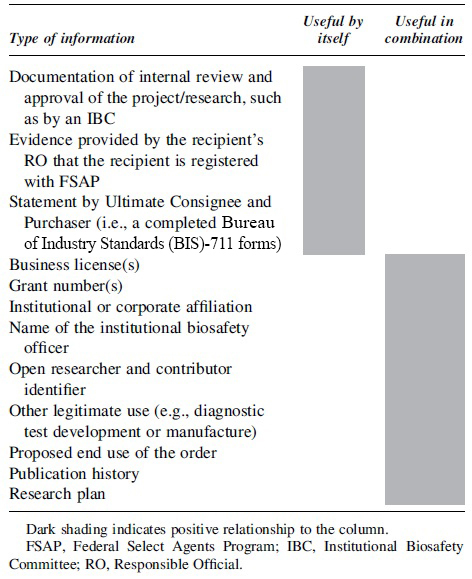

Institutional biosafety officers are well-positioned to aid in verifying the legitimacy of staff at their institution ordering or otherwise receiving materials containing SOCs. They may be able to help confirm several things about their researchers, including their institutional affiliation, Institutional Biosafety Committee review, registration with FSAP, and information about their research plan or legitimate use for those materials.

## Minimizing Burden

According to respondents, implementing the 2010 screening framework is expensive, costing ∼$15/order, and this cost has remained flat over the years whereas the cost of synthesis has decreased. Cost is reportedly driven by the need for a PhD in Bioinformatics to interpret sequence hits. The burden of screening may be expected to increase as the size of databases against which to match BLAST sequences is increasing (i.e., as the definition of SOCs expands beyond solely sequences from regulated pathogens and toxins). Providers indicated that they need new annotated data resources, tools, and approaches to keep biosecurity from becoming a leading component of the per-bp cost, given that nucleic acid synthesis costs have consistently decreased over time.

Most respondents indicated that data retention and records keeping is not a challenge, given ever decreasing data storage costs. However, better guidance would be helpful on what types of customer and sequence data must be retained and what latency is acceptable for retrieval of this information. Highly latent data storage mechanisms are much less costly, especially given customer screening considerations that are also included in the screening framework. The 8-year timeframe for data storage may be a burden for start-up companies, and mitigation processes are necessary in case they are no longer in business after 8 years.

Some respondents asked the U.S. government to provide standardized screening methodologies and a centralized database for screening both sequences and customers—including potential whitelists or restricted lists for customers—as well as clear definitions of the unit of control and of the type of information needed for screening customers. There was also a suggestion that the U.S. government should operate an application programming interface for screening that has a latency for queries of 2 hour or less.

Several respondents also expressed the hope that expanding the scope of the screening framework would not create additional burden if the U.S. government took some of these steps. However, others were nonetheless concerned about potentially increased costs associated with expanding the scope of the screening framework. Support for a cost/benefit approach was voiced to ensure that any additional burden to providers (and customers) can be adequately justified. There were mixed comments regarding whether liability is a concern, with one comment mentioning that the screening framework is perceived as protecting providers from liability.

In response to these concerns the 2023 screening framework recommends that entities selling or otherwise transferring synthetic nucleic acids containing SOCs keep a record of those transfers for at least 3 years. Institutional biosafety officers may be a useful resource for centrally tracking the records of SOC transfers to and from their institutions, such as through the maintenance of material transfer agreements for their researchers. Notably there is no requirement for latency in the retrieval of this information, so lower cost high latency storage of records is an option for entities implementing this policy.

## Technologies Subject to the Guidance

All respondents suggested screening each type of synthetic nucleic acid orders, not just dsDNA. They expressed that this expansion is warranted due to the ease of conversion between single-stranded (ss) DNA, dsDNA, ssRNA, and dsRNA. Additional reasons provided included that modern methodologies allow positive sense ssRNA viral genomes to be transferred directly into cells to produce viruses, and rescue platforms exist for negative sense ssRNA viruses.

Some respondents recommended that the screening framework should apply to the entire synthetic biology supply chain, not just to the providers of synthetic DNA or other oligonucleotides. Also, some responses indicated that benchtop DNA synthesizers pose a serious biosecurity threat, and that significant risks are associated with in-house use of nucleic acid and whole genome synthesizers. In general, comments raised that these devices are increasingly relevant in the U.S. bioeconomy and that a clear risk is associated with them that is as great or greater than the risk from SOCs obtained from providers.

In response to these comments, the recommendation in the 2023 screening framework has been expanded beyond dsDNA to include single- and double-stranded forms of both RNA and DNA. The screening framework also has been expanded to include recommendations for providers, third-party vendors, principal users, and end users as well as manufacturers of benchtop nucleic acid synthesis equipment.

Recommendations for this benchtop equipment also include provisions for the unique challenges associated with both sequence and customer screening in individual laboratories. Institutional biosafety officers may be able to aid in the establishment of user accounts for researchers that have a legitimate and ongoing need to produce nucleic acids containing SOCs, as this has been tasked to institutions in the screening framework.

Also, in response to stakeholder suggestions about emerging technologies, a section has been added on the periodic review, evaluation, and improvement of the guidance. Owing to the continuously evolving nature of synthetic biotechnologies, the guidance includes recommendations that methodologies should be developed to use predictive bioinformatics algorithms to screen sequences that are not Best Matches to any known sequences—especially if no explanation is provided by the customer—to determine whether they could produce proteins that are structurally or functionally identical to SOCs. We intend for this screening framework to be periodically revisited, including by soliciting stakeholder input, and feedback is encouraged from the nucleic acid synthesis industry as well as from their customers.

## Executive Order and Paths Forward

After the publication of the *Screening Framework Guidance for Providers and Users of Synthetic Nucleic Acids* in the *Federal Register* on October 13, 2023, the White House issued an Executive Order on October 30 that included directions to Federal departments and agencies to aid in implementing the recommendations in the screening framework.^18^

Section 4.4 (b) i–iii of the *Executive Order on the Safe, Secure, and Untrustworthy Development and Use of Artificial Intelligence* (AI EO) includes directives to U.S. government departments and agencies to determine standards for conducting and verifying the performance of sequence screening and customer screening, to engage with stakeholders to determine best practices for nucleic acid procurement sequence screening and develop security and access controls for managing SOC databases, develop a stress testing methodology for screening methodologies implemented by synthetic nucleic acid providers, and to establish compliance with the screening framework as a requirement of life sciences research funding.

These directives to departments and agencies of the U.S. government will impact biosafety professionals both domestically and internationally. It is important that the community of biosafety professionals work with researchers at their institutions to help them participate with other entities in the bioeconomy to fulfill the goals of the screening framework and the AI EO. Advances in synthetic biology have brought us to a point where these sequences must be shepherded—by all entities involved in their provision, use, and transfer—throughout the lifetime of the products containing them to ensure the security of the United States and the rest of the world.

Together all entities involved in the provision, use, and transfer of synthetic nucleic acids containing sequences that could threaten public health, agriculture, plants, animals, animal products, or the environment need to work together to ensure that our nation benefits from the great potential of synthetic biology whereas minimizing risks associated with the field.
